# Chronic effects of ambient air pollution on respiratory morbidities among Chinese children: a cross-sectional study in Hong Kong

**DOI:** 10.1186/1471-2458-14-105

**Published:** 2014-02-03

**Authors:** Yang Gao, Emily YY Chan, Liping Li, Patrick WC Lau, Tze Wai Wong

**Affiliations:** 1 Department of Physical Education, Hong Kong Baptist University, Hong Kong, China; 2 School of Public Health and Primary Care, The Chinese University of Hong Kong, Hong Kong, China; 3 Injury Prevention Research Center, Medical College of Shantou University, Shantou, China

**Keywords:** Air pollution, PM_10_, NO_2_, Child, Respiratory morbidity, China, Urban

## Abstract

**Background:**

The chronic health effects from exposure to ambient air pollution are still unclear. This study primarily aims to examine the relationship between long-term exposure to ambient air pollution and respiratory morbidities in Chinese children.

**Methods:**

A cross-sectional study was conducted among 2,203 school children aged 8–10 in three districts with different air pollution levels in Hong Kong. Annual means for ambient PM_10_, SO_2_, NO_2_ and O_3_ in each district were used to estimate participants’ individual exposure. Two questionnaires were used to collect children’s respiratory morbidities and other potential risk factors. Multivariable logistic regression was fitted to estimate the risks of air pollution for respiratory morbidities.

**Results:**

Compared to those in the low-pollution district (LPD), girls in the high-pollution district (HPD) were at significantly higher risk for cough at night (OR_adj._ = 1.81, 95% CI: 1.71-2.78) and phlegm without colds (OR_adj._ = 3.84, 95% CI: 1.74-8.47). In addition, marginal significance was reached for elevated risks for asthma, wheezing symptoms, and phlegm without colds among boys in HPD (adjusted ORs: 1.71-2.82), as well as chronic cough among girls in HPD (OR_adj._ = 2.03, 95% CI: 0.88-4.70).

**Conclusions:**

Results have confirmed certain adverse effects on children’s respiratory health from long-term exposure to ambient air pollution. PM_10_ may be the most relevant pollutant with adverse effects on wheezing and phlegm in boys. Both PM_10_ and NO_2_ may be contributing to cough and phlegm in girls.

## Background

Air pollution is a major environmental health risk in both developing and developed countries [1,2]. It has been estimated that globally each year about 1.3 million premature deaths are attributed to ambient air pollution. In general, people living in less polluted cities have better respiratory and cardiovascular health and the related disease burden can be reduced by improving the ambient air quality [1]. Children are vulnerable to ambient air pollution as they spend more time outdoors, are more physically active, and have higher ventilation rates than adults. Though acute health effects on children’s respiratory morbidities from short-term exposure to air pollution have been well documented, the chronic effects from long-term exposure are still subject to debate. Two Harvard studies revealed chronic adverse effects on children’s cough and bronchitis from exposure to particulate matters (PMs) and sulphur dioxide (SO_2_) [3,4], whilst a Southern California 24-city study reported insignificant associations between PMs and SO_2_ and childhood respiratory morbidities [5]. In addition, this study observed a significant association between nitrogen dioxide (NO_2_) and wheezing in boys [5]. Several studies in Europe and Asia have suggested that traffic-related pollution is significantly associated with children’s asthma, allergic rhinitis, and related symptoms [6-9]. Whilst findings from the International Study of Asthma and Allergies in Childhood (ISAAC) indicated that particulate matters than 10 μm in aerodynamic diameter (PM_10_) was not significantly associated with asthma and rhinoconjunctivities [10].

Hong Kong is located on the coast of Southern China and has a subtropical climate. The ambient air pollution level has improved since 1990, as the result of the implementation of a series of government regulations to restrict fuel’s sulphur content. However, in recent years, progress improvement has slowed down and the air quality has deteriorated, due to the fact that the local air quality is being influenced by the regional air pollution problem of the entire Southern China [11]. Previous studies in the 1990’s have revealed significant adverse effects of ambient air pollution on respiratory morbidities among Hong Kong school children, including cough, phlegm, and asthma [12]. Decreased bronchial hyperactivity among asthmatic children was also observed after a marked reduction in ambient PM_10_ and SO_2_ concentrations [13]. In addition, a four-city study in China showed significant associations between PMs and persistent cough, persistent phlegm, and bronchitis [14]. However, no significant adverse effect was found for SO_2_ and nitrogen oxides (NO_x_) [14]. These Chinese studies were conducted at least a decade ago and air pollution levels at that time were relatively high. Furthermore, none of these studies considered the potential health effects of ozone (O_3_), which is currently a routinely monitored air pollutant in urban China. This study primarily aims to investigate the associations between exposure to ambient air pollution and respiratory morbidities among Chinese primary school children in Hong Kong. In addition, the risks from indoor household factors for respiratory health were also examined.

## Methods

### Study population

This cross-sectional study was conducted from March to June among primary school students in three of Hong Kong. Given that PM_10_ is the major pollutant in Hong Kong and existing evidence suggests that PMs may be the most relevant air pollutant causing children’s respiratory morbidities through long-term exposure [11,15,16], three districts, labelled as low-pollution district (LPD), moderate-pollution district (MPD) and high-pollution district (HPD), were selected from ten districts with urban general air monitoring stations based on the rank of their annual means for PM_10_ over the previous 10 years. LPD and HPD were the districts with the lowest and highest PM_10_ values respectively to maximise the difference in exposure levels. MPD was selected as its PM_10_ annual mean was nearest to the average of those in LPD and HPD. LPD and HPD are new towns with predominantly residential buildings, while MPD is an old urban area with a combination of residential, commercial and factory buildings [11]. Three or four primary schools in each district, which are located within 1 km from the local air monitoring station, were invited to participate in the study using the “the closest to the station, the first to be selected” criterion. In addition, schools with any factories/industrial plants within a 100-meter range were excluded from the study to avoid the influences from point pollution sources. Only Chinese students in grades three and four were invited into the study, as the questionnaires were only in Chinese. In order to reduce exposure misclassification, only students who had been currently living in the district where their school was located for more than 12 consecutive months prior to the study were selected for data analysis. In addition, only children aged 8–10 were included in the data analysis, as few students (3.4%) were beyond this age range.

### Ethics statement

Ethical approval was obtained from the Ethics Committee of the Chinese University of Hong Kong. Informed written consent was obtained from parents or guardians of all participants prior to the study.

### Air pollution data

Four study pollutants, namely PM_10_, NO_2_, SO_2_, and O_3_ are continuously measured in Hong Kong [11]. Annual mean average in the past 10 years (covering the lifetime of the participants) and annual mean in the year prior to the study were used to estimate lifetime and current exposure levels of the participants, respectively [11].

### Questionnaires

Two self-administered questionnaires, *Parent Questionnaire* and *Child Questionnaire* based on the Children’s Questionnaire recommended by the American Thoracic Society (ATS-DLD-78-C), were developed to collect information related to children’s respiratory morbidities and potential confounding factors [17]. Questions to assess asthma-suggesting symptoms were adopted from the Asthma and Allergies in Childhood Written Questionnaire (ISAAC WQ) Chinese version [18]. Parents completed the *Parent Questionnaire* and reported on children’s respiratory symptoms and diseases, socio-demographic characteristics, indoor housing environmental factors, parental history of asthma and allergy, children’s birth weight, birth place and breastfeeding history. Children completed the *Child Questionnaire* and reported smoking habits, time spent outdoors, amount and type of physical activities, participation in team sports and playing with furry toys in the past 12 months.

Respiratory diseases, including asthma, bronchitis and allergic rhinitis, were defined as doctor-diagnosed diseases at any time in the child’s life (*life-time condition*) and in the previous 12 months (*current condition*). Symptoms experienced in the past 12 months included wheezing with shortness of breath, wheezing with medication, any wheezing, cough at night, chronic cough, phlegm with/without colds and sneeze with itchy-watery eyes.

### Statistical analysis

Data were analysed with SPSS for windows 16.0. Percentage and mean and standard deviation (SD) were used to describe the distribution of all variables. Chi-square and one-way ANOVA were performed to compare between-group differences. Multivariable logistic regressions were fitted to estimate the risk of air pollution (independent variable, indicated by district) for respiratory morbidities (dependent variables), after adjustment for other confounders. Adjusted odds ratio (adjusted OR) and 95% confidence intervals (95% CI) were then derived. Age, father’s job, and birth place were forcedly included in all models. Other binary variables were selected in a stepwise method using P < 0.10 and P < 0.15 as entry and removal criteria of variables respectively, including low birth weight (<2.5 kg), breastfeeding, parental asthma, parental allergy, active smoking, physical activity, member of team sports, time spent outdoors, playing with furry toys, having mould in the home, adding new furniture, raising pets, burning incense, passive smoking at home, home more-ventilated in summer and home more-ventilated in winter [19].

## Results

A total of 3,186 children in 11 primary schools were approached and 2,641 (82.9%) participated in the study. More than 95% of the participants (n = 2,534) completed questionnaires and finally 2,203 students (1141 boys and 1062 girls) met our selection criteria and were included in data analyses. Of the 331 students who were excluded from data analysis, 93 were living in other districts (cross-district students), 149 had lived in their school’s district for less than 12 consecutive months, and 89 were below 8 or above 10 years old.

The averages of historical annual means for PM_10_, NO_2_, SO_2_ and O_3_ in the past decade were 48.9 μg/m^3^, 48.4 μg/m^3^, 13.4 μg/m^3^, 38.6 μg/m^3^ in LPD, 55.0 μg/m^3^, 71.0 μg/m^3^, 16.3 μg/m^3^, 22.9 μg/m^3^ in MPD, and 57.6 μg/m^3^, 57.6 μg/m^3^, 18.6 μg/m^3^, 25.8 μg/m^3^ in HPD, respectively. O_3_ concentrations steadily increased over time, whilst no obvious increasing or decreasing trends were observed for the other three pollutants. PM_10_, NO_2_ and SO_2_ were positively correlated with each other, with significant correlations between PM_10_ and SO_2_, and between PM_10_ and NO_2_ (r = 0.70 and 0.51 respectively, P < 0.05). O_3_ was negatively correlated with the other three pollutants, and significantly correlated with PM_10_ and NO_2_ (r = −0.59 and −0.61 respectively, P < 0.05). The negative correlations for O_3_ with PM_10_ and NO_2_ are due to motor vehicles, which is the major source of air pollution in Hong Kong. O_3_ is readily scavenged by reactions with nitric oxide emitted from motor vehicles. In the past 12 months, the annual means for PM_10_, NO_2_, SO_2_ and O_3_ were 55.1 μg/m^3^, 51.4 μg/m^3^, 15.4 μg/m^3^, 42.5 μg/m^3^ in LPD, 56.3 μg/m^3^, 64.7 μg/m^3^, 15.2 μg/m^3^, 35.2 μg/m^3^ in MPD, and 63.8 μg/m^3^, 64.1 μg/m^3^, 22.2 μg/m^3^, 31.7 μg/m^3^ in HPD, respectively. Compared with the historical data, the concentrations of PM_10_ and SO_2_ in LPD, and that of NO_2_ in HPD had increased in the past 12 months towards the levels monitored in MPD.

Table 1 demonstrates the comparison of the participants’ characteristics across districts after stratification by gender. Significant differences were observed in: father’s job, birth place, breastfeeding history, parental allergy, physical activity, member of team sports, playing with furry toys, household mould, pets, burning incense at home, passive smoking, and house ventilation in summer and winter, for single or both genders.

**Table 1:** Characteristics of the subjects

	**Boys (N = 1141)**			**Girls (N = 1062)**		
	**LPD (n = 485)**	**MPD (n = 393)**	** HPD (n = 263)**	**LPD (n = 428)**	**MPD (n = 356)**	**HPD (n = 278)**
***Demographic characteristics***						
Age *(in year) (Mean, SD)*	9.0 *(0.7)*	9.0 *(0.7)*	9.1 *(0.7)*	9.0 *(0.7)*	9.0 *(0.7)*	9.0 *(0.7)*
Blue-collar job of father^a^ (%)	61.4^***^	84.5^***^	79.8^***^	54.9^***^	80.6^***^	81.3^***^
Born in Hong Kong (%)	92.4^***^	77.6^***^	86.7^***^	93.7^***^	75.3^***^	86.0^***^
***Personal & parental characteristics***						
Low birth weight^b^ (%)	10.1	8.1	7.6	9.3	13.2	9.4
Breastfed (%)	27.8^***^	47.5^***^	26.6^***^	27.8^**^	36.2^**^	17.6^**^
Parental asthma (%)	4.5	2.5	5.7	5.4	4.8	7.2
Parental allergy (%)	20.2^**^	13.5^**^	11.8^**^	18.5^*^	12.6^*^	20.1^*^
Active smoking (%)	0.2	1.3	0.8	0.0	0.0	0.0
Physical activity^c^ (%)	41.9^***^	29.3^***^	38.8^***^	39.3^**^	29.8^**^	30.6^**^
Member of team sports (%)	26.4^***^	13.7^***^	25.1^***^	23.6^*^	17.2^*^	23.0^*^
More outdoors^d^ (%)	50.7	48.1	48.7	49.8	50.3	51.8
Playing with furry toys (%)	17.7^*^	16.8^*^	11.0^*^	45.6	42.7	48.2
***Housing environmental characteristics***						
Mould (%)	24.3^***^	21.1^***^	12.9^***^	22.9^#^	22.2^#^	16.5^#^
New furniture (%)	15.9	18.6	16.3	18.7	21.9	18.3
Pets (%)	14.8^**^	11.7^**^	21.3^**^	22.9^***^	13.2^***^	22.3^***^
Incense (%)	20.8^*^	29.0^*^	24.3^*^	17.5	20.5	20.9
Passive smoking (%)	16.5^***^	29.5^***^	24.0^***^	17.8^***^	35.1^***^	30.9^***^
More-ventilated in summer^e^ (%)	63.1^***^	74.8^***^	65.4^***^	61.9^*^	71.6^*^	69.4^*^
More-ventilated in winter^e^ (%)	81.2^***^	76.1^***^	68.4^***^	80.4^***^	72.2^***^	69.8^***^

LPD: low-pollution district; MPD: moderate-pollution district; HPD: high-pollution district.

^#^P < 0.10; *P < 0.05; **P < 0.01; ***P < 0.001.

^a^Including service workers, shop sales workers, skilled workers, unskilled workers, and other kindred workers.

^b^Birth weight less than 2500 g.

^c^Taking part in sports and/or vigorous free play at least three times a week for at least 30 minutes each time.

^d^Indicating that children who spent more hours per week outdoors than the median value for their respective district and gender.

^e^Referring to open windows at night and the whole day during the summer/winter.

Table 2 demonstrates the prevalence of respiratory morbidities by gender and district. Few students (<6.0%) experienced wheezing symptoms, chronic cough and phlegm without colds, in the past 12 months, whilst cough at night, phlegm with colds, and sneeze with itchy-watery eyes were common (20%-32.3%). Few students were diagnosed with asthma, whilst more than 30% of them had allergic rhinitis. In addition, differences across districts reached significance in the following morbidities: cough at night in girls, sneeze with itchy-watery eyes and life-time allergic rhinitis in boys, and phlegm without colds, current and life-time allergic rhinitis in both genders.

**Table 2:** Respiratory symptoms and diseases of the subjects

	**All (%)**				**Boys (%)**						**Girls (%)**					
	**Boys (n = 1141)**		**Girls (n = 1062)**		**LPD (n = 485)**		**MPD (n = 393)**		**HPD (n = 263)**		**LPD (n = 428)**		**MPD (n = 278)**		**HPD (n = 278)**	
***Respiratory symptoms***	n	%	n	%	n	%	n	%	n	%	n	%	n	%	n	%
Wheezing with shortness of breath	1088	2.2	1019	2.4	463	2.2	372	1.6	253	3.2	416	1.9	335	2.1	268	3.4
Wheezing with medication	1088	3.2	1019	3.0	463	3.0	372	2.7	253	4.3	416	2.9	335	3.0	268	3.4
Any wheezing	1088	5.1	1019	4.6	463	5.0	372	4.8	253	5.9	416	3.6	335	4.8	268	6.0
Cough at night	1069	20.1	984	20.6	457	18.6	368	23.4	244	18.0	400	14.8^***^	327	24.5^***^	257	24.9^***^
Chronic cough	1130	4.6	1039	5.0	482	5.6	387	4.1	261	3.4	423	4.0	345	5.2	271	6.3
Phlegm with colds	1053	32.2	983	32.3	456	33.3	358	29.9	239	33.5	402	30.6	322	31.4	259	36.3
Phlegm without colds	1092	6.0	999	5.7	467	5.6^**^	374	4.0^**^	251	10.0^**^	412	3.2^***^	324	5.2^***^	263	10.3^***^
Sneeze with itchy-watery eyes	1098	29.9^#^	1019	26.2^#^	473	34.5^*^	378	25.4^*^	247	27.9^*^	412	29.9^#^	341	25.2^#^	266	21.8^#^
***Respiratory diseases***																
Current asthma	1137	3.2	1056	2.1	484	3.1	391	2.6	262	4.2	424	2.1	354	1.7	278	2.5
Life-time asthma	1137	7.7	1056	6.5	484	7.4	391	7.2	262	8.8	424	6.6	354	5.9	278	7.2
Current bronchitis	1137	11.8	1056	10.6	484	12.8	391	9.7	262	13.0	424	10.1	354	10.5	278	11.5
Life-time bronchitis	1137	20.5	1056	19.4	484	23.8^*^	391	16.9^*^	262	19.8^*^	424	21.5	354	17.2	278	19.1
Current allergic rhinitis	1137	32.7^**^	1056	26.5^**^	484	36.8^**^	391	26.6^**^	262	34.4^**^	424	33.3^***^	354	18.4^***^	278	26.6^***^
Life-time allergic rhinitis	1137	37.6^**^	1056	31.7^**^	484	41.9^*^	391	32.0^*^	262	38.2^*^	424	38.4^***^	354	22.9^***^	278	32.7^***^

LPD: low-pollution district; MPD: moderate-pollution district; HPD: high-pollution district.

^#^P < 0.10; ^*^P < 0.05; ^**^P < 0.01; ^***^P < 0.001.

Tables 3 and 4 present the risks of ambient air pollution for respiratory morbidities after stratification by gender and adjustment for other risk factors. Compared to those in LPD, girls in HPD were at significantly higher risk for cough at night and phlegm without colds (OR_adj._ = 1.81, 95%CI = 1.17-2.78; and OR_adj._ = 3.84, 95%CI = 1.78-8.47, P < 0.05), and girls in MPD were at significantly higher risk for cough at night only (OR_adj._ = 1.65, 95%CI = 1.08-2.52, P < 0.05). Contrary to our hypothesis, both boys and girls in MPD were significantly less likely to have current and life-time allergic rhinitis compared to their counterparts in LPD (adjusted ORs ranged from 0.71 to 0.56, P < 0.05). In addition, boys in HPD were found to be at marginally significant higher risk for wheezing symptoms, phlegm without colds, and current and life-time asthma compared to those in LPD (adjusted ORs ranged from 1.71 to 2.82, P < 0.10). Marginal significance for elevated risk for cough at night was also found among boys in MPD (OR_adj._ = 1.43, 95%CI = 0.98-2.08, P < 0.10).

**Table 3:** Adjusted odds ratios (ORs) and 95% CIs of air pollution and other risk factors for respiratory symptoms by gender

	**Wheezing with shortness of breath**	**Wheezing with medication**	**Any wheezing**	**Cough at night**	**Chronic cough**	**Phlegm with colds**	**Phlegm without colds**	**Sneeze with itchy-watery eyes**
***Boys***								
MPD	0.64	0.80	0.86	1.43^#^	0.93	0.88	0.66	0.75
*(LPD as ref)*	*(0.16, 2.60)*	*(0.28, 2.35)*	*(0.36, 2.05)*	*(0.98, 2.08)*	*(0.46, 1.87)*	*(0.62, 1.26)*	*(0.34, 1.30)*	*(0.53, 1.06)*
HPD	2.82^#^	2.47^#^	2.09^#^	1.12	0.83	1.25	1.71^#^	0.85
*(LPD as ref)*	*(0.95, 8.34)*	*(0.98, 6.21)*	*(0.93, 4.69)*	*(0.72, 1.74)*	*(0.37, 1.89)*	*(0.85, 1.82)*	*(0.94, 3.10)*	*(0.58, 1.24)*
Low birth weight					3.27^**^			
					*(1.48, 7.25)*			
Physical activity						0.77^#^		
						*(0.57, 1.04)*		
Member of team sports		0.30^#^	0.21^*^					
		*(0.09, 1.05)*	*(0.06, 0.70)*					
Mould	3.18^*^	2.82^*^	2.24^*^	1.83^***^		1.55^*^		1.32
	*(1.19, 8.54)*	*(1.24, 6.39)*	(1.10, 4.56)	*(1.27, 2.64)*		*(1.11, 2.19)*		*(0.94, 1.84)*
Passive smoking			1.90^#^			1.31		
			*(0.92, 3.93)*			*(0.92, 1.86)*		
More-ventilated in summer								0.68^*^
								*(0.51, 0.92)*
More-ventilated in winter	3.25							
	*(0.71, 14.9)*							
***Girls***								
MPD	0.75	0.75	1.68	1.65^*^	1.16	1.05	1.44	0.81
*(LPD as ref)*	*(0.15, 3.68)*	*(0.23, 2.43)*	*(0.68, 4.17)*	*(1.08, 2.52)*	*(0.48, 2.82)*	*(0.72, 1.53)*	*(0.58, 3.54)*	*(0.55, 1.19)*
HPD	1.01	1.00	1.53	1.81^**^	2.03^#^	1.36	3.84^***^	0.75
*(LPD as ref)*	*(0.24, 4.34)*	*(0.32, 3.10)*	*(0.61, 3.86)*	*(1.17, 2.78)*	*(0.88, 4.70)*	*(0.93, 1.99)*	*(1.74, 8.47)*	*(0.50, 1.12)*
Low birth weight	4.54^*^				2.42^*^			
	*(1.25, 16.4)*				*(1.02, 5.74)*			
More outdoors				1.44^*^	1.70			
				*(1.03, 2.01)*	*(0.84, 3.43)*			
Mould					3.28^***^	1.77^***^	1.79	2.33^***^
					*(1.65, 6.52)*	*(1.25, 2.51)*	*(0.89, 3.58)*	*(1.65, 3.31)*
Pets		0.17^#^						
		*(0.02, 1.31)*						
New furniture				1.56^*^				
				*(1.05, 2.31)*				
Incense					2.10^*^			1.41^#^
					*(1.01, 4.36)*			*(0.96, 2.07)*
Passive smoking				1.46^*^			2.32^*^	
				*(1.01, 2.10)*			*(1.19, 4.51)*	
More-ventilated in winter		6.65^#^						
		*(0.86, 51.1)*						

LPD: low-pollution district; MPD: moderate-pollution district; HPD: high-pollution district.

^#^P < 0.10; ^*^P < 0.05; ^**^P < 0.01; ^***^P < 0.001.

District (indicating air pollution), age, father’s occupation, birth place were forcibly included in the models. Other factors were selected using the stepwise method and the entry and removal criteria of variables were P = 0.10 and P = 0.15, respectively.

The variables presented with adjusted ORs and 95% CIs were included in the final models, and the remaining variables (with adjusted ORs only) were adjusted for the variables included in the final models only.

Parental asthma was adjusted for in all models of wheezing symptoms (adjusted ORs: 2.44-6.62, P < 0.05); Parental allergy was adjusted for in all models except for wheezing symptoms among boys, phlegm without colds among both genders (adjusted ORs: 1.48-4.07, P < 0.05).

**Table 4:** Adjusted odds ratios (ORs) and 95% CIs of air pollution and other risk factors for respiratory diseases by gender

	**Current asthma**	**Life-time asthma**	**Current bronchitis**	**Life-time bronchitis**	**Current allergic rhinitis**	**Life-time allergic rhinitis**
***Boys***						
MPD *(LPD as ref)*	0.87	1.23	0.85	0.74	0.69^*^	0.71^*^
	*(0.31, 2.40)*	*(0.65, 2.31)*	*(0.52, 1.39)*	*(0.50, 1.08)*	*(0.49, 0.98)*	*(0.50, 0.99)*
HPD *(LPD as ref)*	2.26^#^	1.82^#^	1.22	1.00	1.07	1.03
	*(0.91, 5.61)*	*(0.95, 3.49)*	*(0.73, 2.02)*	*(0.67, 1.51)*	(0.74, 1.54)	*(0.72, 1.48)*
Member of team sports	0.29^#^					
	*(0.09, 1.01)*					
Playing with furry toys					1.44^#^	1.57^*^
					*(0.99, 2.09)*	*(1.08, 2.28)*
Mould	5.32^**^	2.26^**^				
	*(1.82, 15.56)*	*(1.31, 3.90)*				
Incense					1.47^*^	1.52^*^
					*(1.05, 2.05)*	*(1.09, 2.12)*
More-ventilated in summer					0.72^*^	0.70^*^
					*(0.53, 0.97)*	*(0.52, 0.95)*
More-ventilated in winter			0.62^*^		1.65^**^	1.50^*^
			*(0.41, 0.96)*		*(1.15, 2.36)*	*(1.06, 2.12)*
***Girls***						
MPD *(LPD as ref)*	1.40	0.87	1.26	0.93	0.56^**^	0.59^**^
	*(0.39, 5.04)*	*(0.43, 1.77)*	*(0.74, 2.15)*	*(0.61, 1.41)*	*(0.38, 0.82)*	*(0.41, 0.85)*
HPD *(LPD as ref)*	0.85	0.91	1.05	0.89	0.78	0.89
	*(0.20, 3.69)*	*(0.44, 1.88)*	*(0.60, 1.85)*	*(0.58, 1.37)*	*(0.53, 1.15)*	*(0.62, 1.27)*
Physical activity		1.91^*^				
		*(1.08, 3.38)*				
More outdoors		0.46^*^				
		*(0.25, 0.83)*				
Mould					1.38^#^	1.29
					*(0.97, 1.98)*	*(0.92, 1.82)*
Pets		0.32^*^				
		*(0.12, 0.84)*				

LPD: low-pollution district; MPD: moderate-pollution district; HPD: high-pollution district.

^#^P < 0.10; ^*^P < 0.05; ^**^P < 0.01.

District (indicating air pollution), age, father’s occupation, birth place were forcibly included in the models. Other factors were selected using stepwise method and the entry and removal criteria of variables were P = 0.10 and P = 0.15, respectively.

The variables presented with adjusted ORs and 95% CIs were included in the final models, and the remaining variables (with adjusted ORs only) were adjusted for the variables included in the final models only.

Parental asthma was adjusted for in all models except for current and life-time allergic rhinitis among girls (adjusted ORs: 1.89-5.32, P < 0.05); Parental allergy was adjusted for in all models except for current asthma among boys (adjusted ORs: 2.30-4.33, P < 0.05).

In the multivariable analysis (Tables 3 and 4), age, father’s job and birth place were adjusted for in all models no matter their significance. Blue-collar job of father was significantly and negatively associated with phlegm without colds and life-time allergic rhinitis for girls (adjusted ORs ranged from 0.56 to 0.71, P < 0.05). Boys born in Hong Kong were significantly less likely to have cough at night (OR_adj._ = 0.63, 95%CI = 0.40-0.98, P < 0.05), but significantly more likely to suffer from sneeze with itchy-watery eyes, and allergic rhinitis (adjusted ORs ranged from 1.80 to 2.27, P < 0.05). Parental asthma and parental allergy were risk factors for most respiratory conditions (adjusted ORs ranged from 1.89 to 5.32, P < 0.05). In terms of the other confounders, physical activity, being a member of a sports team, raising pets, and more-ventilated in summer were significantly and negatively associated with some respiratory conditions; whilst children with low birth weight, spending more time outdoors, playing with furry toys, having mould at home, new furniture, burning incense, passive smoking and more-ventilated in winter were significantly more vulnerable to some morbidities (Tables 3 and 4). It is worth noting that more-ventilated in summer seemed to be a protective factor for allergic rhinitis, and sneeze with itchy-watery eyes, whilst more-ventilated in winter had adverse effects on allergic rhinitis. In addition, being physically active was a significantly high risk factor for life-time asthma in girls.

We hypothesised that exposure to current air pollution (in the past 12 months) would have more influence on respiratory symptoms and diseases (in the past 12 months), when compared to historical exposure. Thus, the average monthly means for the air pollutants over the past 12 months allowed us to estimate the associations between some individual pollutants and current symptoms and diseases through regrouping the districts as follows: 1) LPD and MPD were combined together and then compared with HPD, as PM_10_ in LPD and MPD were both at a low level (55.1 μg/m^3^ and 56.3 μg/m^3^ respectively); 2) MPD and HPD were combined and then compared with LPD to reflect the same high level of NO_2_ in MPD and HPD (64.7 μg/m^3^ and 63.8 μg/m^3^ respectively). Significant or marginally significant adjusted ORs and their 95% CIs after regrouping are presented in Table 5. Boys in HPD were at significantly higher risk for wheezing symptoms and phlegm without colds compared to those in the combined LPD and MPD (adjusted ORs ranged from 2.03-2.76, P < 0.05), suggesting that it was PM_10_, but not NO_2_, which was mainly responsible for these adverse effects. However, both PM_10_ and NO_2_ contributed to the adverse effects on cough and phlegm in girls, as shown in Table 5 (adjusted ORs ranged from 1.71-2.87, P < 0.05). Contrary to our hypothesis, NO_2_ was significantly and negatively associated with current allergic rhinitis in girls.

**Table 5:** Adjusted odds ratios (ORs) of individual pollutants for respiratory conditions by gender after regrouping districts (significant or marginally significant only)

	**Adjusted OR**	***95% CI***	**P**
**High PM**_**10**_^**a**^			
***Boys***			
Wheezing with shortness of breath	2.76	*(1.07, 7.13)*	0.036
Wheezing with medication	2.71	*(1.20, 6.15)*	0.017
Any wheezing	2.17	*(1.09, 4.34)*	0.028
Phlegm without colds	2.03	*(1.19, 3.44)*	0.009
Current asthma	2.03	*(0.93, 4.45)*	0.076
***Girls***			
Cough at night	1.38	*(0.96, 1.99)*	0.083
Phlegm without colds	2.87	*(1.56, 5.27)*	0.001
**High NO**_**2**_^**b**^			
***Girls***			
Cough at night	1.71	*(1.18, 2.49)*	0.005
Phlegm without colds	2.17	*(1.08, 4.34)*	0.029
Current allergic rhinitis	0.65	*(0.47, 0.89)*	0.008

^a^the risk of high PM_10_ was estimated by comparing high-pollution district (HPD) to the combination of low-pollution district (LPD) and moderate-pollution district (MPD);

^b^the risk of high NO_2_ was estimated by comparing the combination of MPD and HPD to LPD;

Confounders adjusted were the same as those included in the models without regrouping districts.

## Discussion

We conducted a cross-sectional study among 2,203 Hong Kong primary school students aged 8–10 years and uncovered certain adverse effects of ambient air pollution on respiratory symptoms and diseases. Girls living in HPD were at significantly higher risk for cough at night and phlegm without colds. Marginal significance for elevated risks was found for wheezing symptoms, phlegm without colds, and asthma in HPD boys, as well as chronic cough in HPD girls. Both genders in MPD were significantly less likely to have allergic rhinitis. Individual pollutant analysis further revealed that PM_10_ was the major pollutant responsible for the adverse effects on wheezing and phlegm in boys, whilst both PM_10_ and NO_2_ were contributing to cough and phlegm in girls.

Our findings confirmed similar adverse effects of air pollution in several one-city cross-sectional studies in China [12,20-25]. Higher prevalence rates of wheezing, cough, phlegm and asthma were consistently found among children living in high pollution areas, though outcome indicators across the studies were not exactly the same. In a previous study in Hong Kong, Yu et al. revealed that children living in a high pollution district were at high risk for frequent cough, frequent phlegm and asthma, but not for wheezing, bronchitis and allergic rhinitis [12]. Marginal significance was found for high risks for chronic cough and wheezing with shortness of breath [12]. In Yu’s study, the risk of ambient air pollution was estimated without further breakdown by gender and therefore the potential gender difference in response to exposure was unclear. In addition, the air pollutants assessed in the previous study consisted of only PM_10_, SO_2_, and NO_2_. The differences in the annual mean concentrations of the three pollutants between high and low pollution districts in Yu’s study were larger than those between HPD and LPD in the current study.

In this study, further individual pollutant analyses suggests that PM_10_ was mainly responsible for the elevated risk for some respiratory morbidities in children (including wheezing for boys, cough for girls, and phlegm for both genders), whilst NO_2_ could negatively affect cough and phlegm for girls only. PM_10_ is a mixture of solid, liquid or solid and liquid particles, and a large body of chemicals within PM_10_ (e.g. sulphates, polycyclic aromatic hydrocarbons, metals) can solely, or in combination, cause biological harm, which gives scientific support for its stronger associations with respiratory morbidities than the other pollutants observed in this study. Similar to our results, two previous cross-sectional studies in China, examining the health effects of individual pollutants, found that PMs were more responsible for the adverse effects than other pollutants [14,26]. A four-city study in China reported a significantly adverse effect of PM_10_ on phlegm, whilst the effects of NO_x_ and SO_2_ were weak and insignificant [14]. A six-city study in Northern China (Liaoning Province) found that total suspended particles (TSP), NO_x_ and SO_2_ were significantly and positively associated with cough, phlegm, and asthma in single pollutant models, whilst the adverse effects of SO_2_ on all morbidities and that of NO_x_ on asthma decreased and were no longer significant in three-pollutant models, suggesting TSP was a stronger pollutant than the two other gaseous pollutants [26]. A meta-analysis involving four cross-sectional studies in China, examining the relationship between long-term exposure to ambient air pollution and child respiratory symptoms and diseases, indicated that an increase of per μg/m^3^ annual mean for PM_10_ could increase the likelihood of respiratory morbidity by 0.44% (S.E. 0.02) [27]. It is noteworthy that, in our study, the results of the individual pollutant analysis (Table 5) should be considered cautiously, as we were unable to remove the potential effects of other pollutants from that of the individual pollutant. For instance, the combination of the two low PM_10_ districts (LPD + MPD) also had lower levels of SO_2_ compared to HPD, which limited our ability to distinguish the health effects of PM_10_ from SO_2_. Similarly, the combination of the two high NO_2_ districts (MPD + HPD) was also low in O_3,_ if taking O_3_ into consideration.

Contrary to our hypothesis, we found significantly negative associations between air pollution and allergic rhinitis. Compared to LPD, children of both genders in MPD were less likely to suffer from allergic rhinitis. After regrouping the districts, results from the further individual pollutant analysis, showed that children in the high NO_2_ but low O_3_ districts (MPD + HPD) were less likely to suffer from allergic rhinitis than those in the low NO_2_ but high O_3_ district (LPD) in girls only (Table 5). It is biologically impossible that exposure to high NO_2_ could be of benefit to health. Thus, the high O_3_ in LPD might have contributed to the negative associations. In our study, O_3_ was negatively correlated with the three other pollutants due to the fact that background O_3_ in more polluted areas can be removed through reacting with NO [11]. In line with our study, a cross-sectional study in 331,686 middle-school students in Taiwan reported that allergic rhinitis was positively associated with long-term exposure to O_3_[10]. However, other studies failed to find any chronic adverse effects of O_3_ on respiratory morbidities (including allergic rhinitis) in school children [5,28]. Unlike acute effects, the chronic effect of O_3_ has not yet been well documented. One possibility is that the strong associations between PMs and respiratory morbidities in many studies may make it difficult to assess the individual role of ozone *per se*[2].

The gender-different responses to air pollution and greater NO_2_ effects on girls observed in our study were consistent with some previous studies [29-31], while other studies reported no gender differences [9], or greater effects of NO_2_ on boys [5]. Children with fathers in blue-collar jobs in this study were less likely to suffer from phlegm without colds and allergic rhinitis, which confirmed the findings from another study in Hong Kong [12]. Children born in Hong Kong were at higher risk for allergic rhinitis, reflecting the fact that the prevalence of allergic rhinitis was higher in Hong Kong Children than those in other places of China [12,18]. Low birth weight was a risk factor for wheezing with shortness of breath and chronic cough, which is consistent with results from a study in China [26]. Children spending more time outdoors are expected to be exposed to more air pollution, however we only found an adverse relationship with cough at night in girls. In terms of housing environmental factors, we found that mould, passive smoking, burning incense, and adding new furniture were positively associated with some respiratory morbidities, in line with the findings from many other studies around the world. A more ventilated home in winter was a risk factor, whilst a more ventilated home in summer was a protective factor for respiratory morbidities. One possibility is that ambient air pollution in winter was heavy; thus, more ventilation might increase the exposure level to ambient air pollution, and therefore increase the likelihood of respiratory morbidities. However, outdoor air quality in summer was relatively clear, and therefore good ventilation could improve the indoor air quality and reduce the risks for respiratory symptoms and diseases. Some factors in our study were observed to have opposite associations with respiratory conditions, contrary to our hypothesis, such as being a team sport member for current asthma in boys, and pet keeping for life-time asthma in girls. It is possible that asthmatic children might not join in any team sports, and families with asthmatic children might avoid keeping pets, because parents of asthmatic children in Hong Kong are well aware of these two risk factors.

This study is subject to several limitations. It is a cross-sectional study, and therefore we are unable to establish the temporal order of cause and effect. Exposure misclassification may inevitably exist and bias our findings most likely towards the null, like other studies using ambient air monitoring data as a proxy of personal exposure levels [32]. As individual exposures among children living in the same district can vary largely due to influences from diverse micro-environmental and personal factors, such as different air pollution levels at home and different physical activity levels. Though we controlled for several potential household and behavioural factors, the bias could not be entirely corrected. Recall bias may be unavoidable with self-reported retrospective data. For example, parents were more likely to recall severe respiratory conditions than mild ones, and recent episodes than older ones. Such bias may have introduced misclassification of diseases and resulted in underestimations of the adverse health effects [32]. In this study, we only recruited Chinese students, although overall less than 1% of students in Hong Kong mainstream primary schools are non-Chinese. However, our results are unable to reflect the situation of non-Chinese children in Hong Kong. The length of residual time of the participants may affect the associations between air pollution and respiratory morbidities, especially for life-time diseases. However, we did not collect such data, and therefore could not control for its influence. If students in HPD had ever lived in a less polluted district than HPD, and/or students in LPD had ever lived in a more polluted district than LPD, the health risks of air pollution in this study might have been underestimated, and *vice versa*. Unlike those in western countries, most families having children in Hong Kong would not prefer to move in order to create a stable environment for their children’s growth. As shown in this study, only 5.6% of the participants (149/2641) had ever moved from one district to another over the past 12 months. Thus, the confounding effect of the length of time living in an area might not be a major threat to this study. In the future, there is a need for cohort studies to confirm the associations between air pollution and children’s respiratory morbidities observed in this study. In addition, the relatively small differences in air pollution level across the three study districts limits our ability to test significant effects of air pollution on children’s respiratory morbidities. Further studies should enlarge the range of air pollutant concentrations via multi-city studies. There were only three districts in this study; we were therefore unable to distinguish health effects of individual pollutant (e.g. PM_10_) from those of other pollutants.

## Conclusions

In conclusion, we have confirmed certain adverse effects on child respiratory health from exposure to ambient air pollution in a Chinese urban setting. The adverse effects observed for PM_10_ appear to be stronger than other pollutants. Girls were more vulnerable to NO_2_ than boys. Our findings may be transferable to other developing countries characterized by similar air pollution patterns to China. Along with the publicity of air quality monitoring data and the introduction of PM_2.5_ as a routine monitoring air pollutant in some big Chinese cities in 2012, the relatively high level of ambient air pollution and its health impact have been recently received unprecedented attentions from the Chinese public. There is an urgent call for the government to take effective actions to reduce air pollution level. Our findings provide some evidence for professionals and policy-makers to improve air quality, in particular PM_10_ and NO_2_, to protect the health of children.

## Abbreviations

95% CI: 95% confidence intervals; ATS-DLD-78-C: Children’s Questionnaire recommended by the American Thoracic Society; HKEPD: Environmental Protection Department of Hong Kong; HPD: High-pollution district; ISAAC: International Study of Asthma and Allergies in Childhood; ISAAC WQ: Asthma and Allergies in Childhood Written Questionnaire; LPD: Low-pollution district; MPD: Moderate-pollution district; NO2: Nitrogen dioxide; NOx: Nitrogen Oxides; O3: Ozone; OR: Odds ratio; PM10: Particulate matters less than 10 μm in aerodynamic diameter; SD: Standard deviation; SO2: Sulphur dioxide.

## Competing interests

All authors declare that they have no competing interests.

## Authors’ contributions

YG conceived the idea of the study, prepared the study proposal, collected data in the field, performed the data analysis, and drafted the manuscript. EYYC and LL participated in data analysis and assisted with the interpretation of data, and critically reviewed the manuscript. PWCL critically reviewed the manuscript. TWW conceived the study idea, supervised the preparation of the study proposal, data collection, data analysis, interpretation of data, and critically reviewed the manuscript. All authors read and approved the final manuscript. All authors participated in critical appraisal and revision of the manuscript.

## Pre-publication history

The pre-publication history for this paper can be accessed here:

http://www.biomedcentral.com/1471-2458/14/105/prepub
